# The interactive effects of arbuscular mycorrhiza and plant growth-promoting rhizobacteria synergistically enhance host plant defences against pathogens

**DOI:** 10.1038/s41598-017-16697-4

**Published:** 2017-11-27

**Authors:** Alejandro Pérez-de-Luque, Stefanie Tille, Irene Johnson, David Pascual-Pardo, Jurriaan Ton, Duncan D. Cameron

**Affiliations:** 1 Department of Animal and Plant Sciences, Alfred Denny Building, Plant Production and Protection (P3) Centre, University of Sheffield, Western Bank, Sheffield, S10 2TN UK; 2 IFAPA, Centro Alameda del Obispo, Área de Mejora y Biotecnología, Avda. Menédez Pidal s/n, Apdo. 3092, 14004 Córdoba, Spain

## Abstract

Belowground interactions between plant roots, mycorrhizal fungi and plant growth-promoting rhizobacteria (PGPR) can improve plant health via enhanced nutrient acquisition and priming of the plant immune system. Two wheat cultivars differing in their ability to form mycorrhiza were (co)inoculated with the mycorrhizal fungus *Rhizophagus irregularis* and the rhizobacterial strain *Pseudomonas putida* KT2440. The cultivar with high mycorrhizal compatibility supported higher levels of rhizobacterial colonization than the low compatibility cultivar. Those levels were augmented by mycorrhizal infection. Conversely, rhizobacterial colonization of the low compatibility cultivar was reduced by mycorrhizal arbuscule formation. Single inoculations with *R. irregularis* or *P. putida* had differential growth effects on both cultivars. Furthermore, while both cultivars developed systemic priming of chitosan-induced callose after single inoculations with *R. irregularis* or *P. putida*, only the cultivar with high mycorrhizal compatibility showed a synergistic increase in callose responsiveness following co-inoculation with both microbes. Our results show that multilateral interactions between roots, mycorrhizal fungi and PGPR can have synergistic effects on growth and systemic priming of wheat.

## Introduction

The plant immune system can be primed in response to specific signals, released either by biological or chemical agents^1^. This priming of defences provides plants with an augmented capacity to express basal resistance^2^, enabling a faster and stronger defensive response against pathogen attack. Symbiotic microorganisms such as arbuscular mycorrhizal fungi (AMF) and plant growth-promoting rhizobacteria (PGPR) can induce systemic resistance to both aerial and soil borne pathogens^3–6^. Moreover, the presence of both AMF and PGPR in the rhizosphere is known to be an important determinant of plant health in general^7–9^, that is, of the ability of a plant to carry out its physiological functions to the best of its genetic potential.

The regulation of root microbiome structure, and hence any beneficial effect on the plant, is extremely complex. Apart from environmental conditions (weather, soil nutrient status and physical structure, etc.), interactions between mycorrhizal fungi (from the Glomeromycota phylum), soil bacteria (from several genera such as *Pseudomonas*, *Azotobacter*, *Bacilus*, *Azospirillum*, etc.) and the plant play a crucial role shaping the microbiome community^8,10^. For example, host plant genotype strongly influences the extent to which AMF and PGPR colonize the host roots^11–13^. This effect is generally exerted through differences in the profile of plant metabolites exuded by the root that can attract specific organisms to the rhizosphere. For example, strigolactones are known to play a key role in recruiting AMF^14^ and benzoxazinoids have been shown to induce positive chemotaxis in the case of the PGPR *Pseudomonas putida* ^15^.

It can be misleading, however, to consider the chemistry of root exudation, and the signals encoded within, in isolation of the organisms that respond to those signals. This is because the interaction of a specific microorganism with the plant can alter the production of root metabolites^16,17^, consequently influencing the composition chemoattractants in the resultant root exudates. For example, AMF are responsible for what is known as the mycorrhizosphere effect: the enhanced microbial activity surrounding mycorrhizal roots^16,18,19^.

In this context, the priming of defences or the induction of resistance could be partially achieved by the combined action of both AMF and PGPR. Support for this hypothesis comes from the recent observation that AMF can enhance the accumulation of the benzoxazinoid, DIMBOA, in plant roots^20,21^ and thus may amplify positive chemotaxis by the PGPR *P. putida*, because benzoxazinoids act as semiochemicals for this bacteria^15^. As a consequence of this discovery, Cameron *et al*.^10^ proposed a model to explain how both AMF and PGPR could act together to define the Mycorrhiza-Induced Resistance (MIR), the phenomenon of AM induced protection against biotic stress^22^. This model postulates that in the earliest phase, the plant root exudes strigolactones that are known to facilitate increased AMF colonisation of the roots^14,23^. The plant immune system locally responds to the first stages of AMF colonisation, involving a transient activation of salicylic acid (SA)-dependent defences that can prime systemic plant tissues for this type of defence^24^. In order for the AMF to form a stable symbiosis, the fungus must locally supress these plant defences via hitherto unknown effectors^25^. Reprogramming of local plant defences, probably including induction of the SA-antagonist abscisic acid (ABA)^26^, results in changes to the composition of subsequent root exudates and possibly systemic priming of ABA-dependent defences. Finally, during the last phase, the mycorrhizosphere has established and recruited rhizobacteria that can systemically prime jasmonic acid (JA)- and ethylene-dependent defences^27^. In this way, the immune priming appears as a more complex process that involves a spatio-temporal interplay of different rhizosphere organisms (e.g. mycorrhiza and rhizobacteria) and corresponding host reactions.

The aim of the current study is starting to unravel these complex interactions and test the predictions of the Cameron *et al*.^10^ model by investigating: (i) the responses of plant growth to monoxenic colonisation by the plant-beneficial rhizobacterium *P. putida* KT2440 and the AMF species *Rhizophagus irregularis* (syn. *Glomus intraradices*) as well as under co-inoculated conditions; (ii) the effects of *R. irregularis* on the colonisation of the rhizoplane by *P. putida* (and *vice versa*); (iii) the degree of immune priming of plant tissues (assessed by callose deposition as a proxy) under monoxenic colonisation by *P. putida* KT2440 or *R. irregularis* as well as under co-inoculated conditions; (iv) the influence of wheat genotype on the root colonisation by *P. putida* KT2440 and *R. irregularis*

## Results

### AMF colonisation

**Figure 1:**
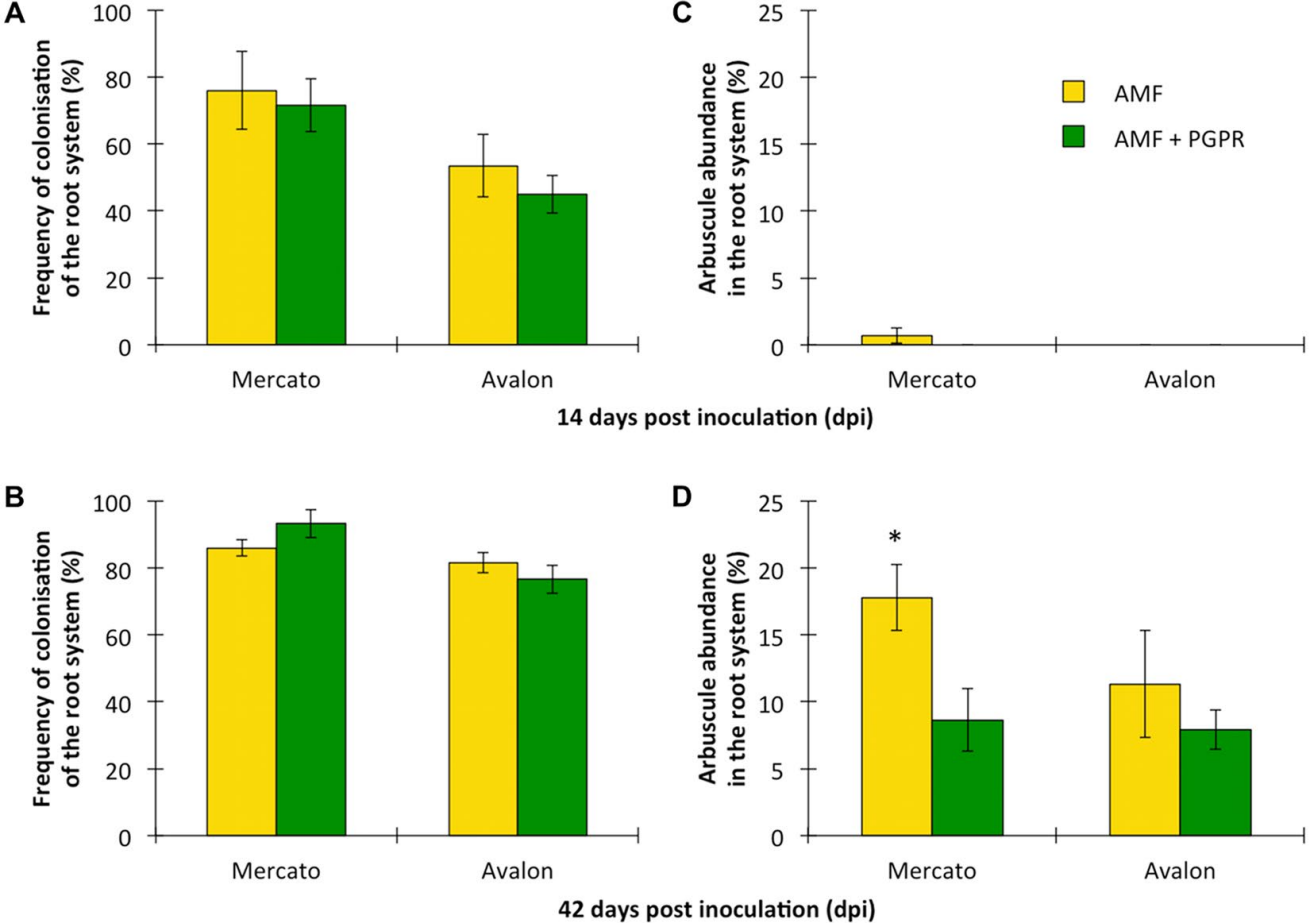
Estimation of colonisation of wheat roots by *Rhizophagus irregularis* according to Trouvelot *et al*.^28^. Two indices were used: frequency of colonisation of the root system (F) at 14 (**A**) and 42 (**B**) days post inoculation, and arbuscule abundance in the root system (**A**) at 14 (**C**) and 42 (**D**) days post inoculation. Asterisk indicates significant differences within the same cultivar (Tukey; P < 0.05). Shown are average values (n = 10; ±standard error).

Colonisation of wheat roots by *R. irregularis* was estimated by calculation of two frequently used indices^28^: frequency of colonisation of the root system (F) and arbuscule abundance in the root system (A). Regarding frequency of colonisation, the cultivar ‘Mercato’ (which exhibited higher mycorrhizal colonisation), contained more fungal hyphae per unit root length at 14 days than the cultivar ‘Avalon’ (which exhibited lower mycorrhizal colonisation) (Fig. 1A). By 42 days, there was no difference in the fungal hyphae per unit root length between either cultivar (Fig. 1B). Co-inoculation with *P. putida* KT2440 did not affect the amount of fungal hyphae per unit root length in either cultivar at either time point.

A differential response was also found when arbuscule abundance was measured with virtually no arbuscules being observed in either cultivar at 14 days (Fig. 1C). However by 42 days arbuscules were much more frequently observed with ‘Mercato’ significantly containing a 60% more number of arbuscules per unit root length when compared with ‘Avalon’ (Fig. 1D). At 42 days, *P. putida* KT2440 significantly reduced the number of arbuscules per unit root length by half in ‘Mercato’. A reduction in number of arbuscules per unit root length was also observed in ‘Avalon’ but this was not significant.

### PGPR colonisation

**Figure 2:**
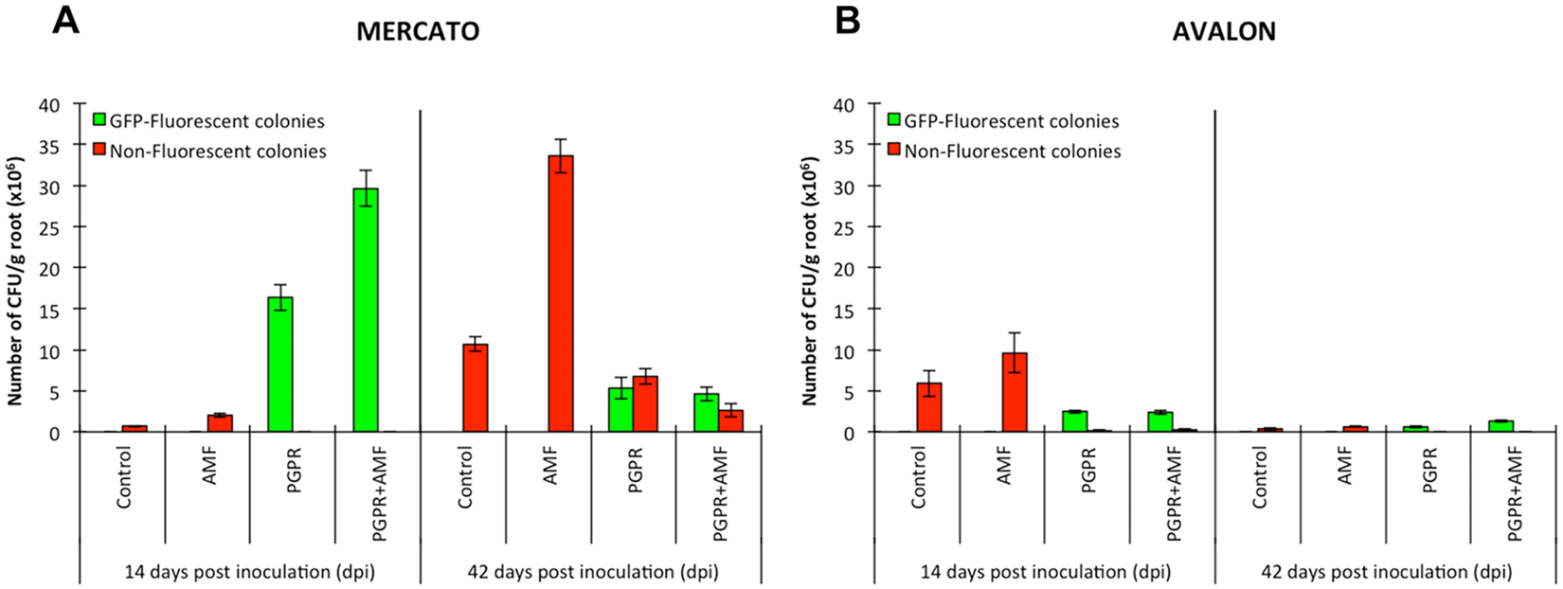
Estimation of bacterial colonisation of wheat roots by *Pseudomonas putida* KT2440 and other spontaneous bacteria. (**A**) Number of colony forming units (CFU) per gram of root of wheat cultivar ‘Mercato’ at 14 and 42 days post inoculation. Green bars correspond to GFP-tagged *P. putida* KT2440 colonies and red bars to unidentified spontaneous growing bacteria. (**B**) Idem as (**A**) but for wheat cultivar ‘Avalon’. Shown are average values (n = 10; ±standard error).

In the cultivar ‘Mercato’, there were significantly more culturable bacteria on the rhizoplane of *R. irregularis*-infected plants in comparison to non-mycorrhizal plants at 14 days (Fig. 2A, Table 1). By contrast, no other bacteria were detected in the rhizoplane when GFP-tagged *P. putida* KT2440 was added to the growth substrate. At 14 days, *P. putida* KT2440 cells were recovered from the rhizoplane in high numbers after being added to the rhizotron at day 0, which was significantly increased by the presence of *R. irregularis*. By 42 days, the bacterial titre of *P. putida* KT2440 had declined dramatically, and was significantly lower than numbers of other (non-GFP expressing) culturable bacteria on the rhizoplane of ‘Mercato’. Similarly as for *P. putida* KT2440, the titre of these other culturable bacteria was significantly enhanced by the presence of *R. irregularis* when *P. putida* KT2440 was absent. Although rhizoplane colonization by *P. putida* KT2440 decreased significantly over time, the proportion of *P. putida* KT2440 remained significantly high for mycorrhizal roots of ‘Mercato’ relative to the other non-GFP-expressing bacteria at 42 days.

**Table 1:** Two-way ANOVA of bacterial colonization.

MERCATO								
	14 days post inoculation				42 days post inoculation			
	Factor	d.f.	F	P	Factor	d.f.	F	P
GFP-fluorescent colonies	AMF	1	24.47	<0.001	AMF	1	0.85	0.358
	PGPR	1	290.86	<0.001	PGPR	1	17.06	<0.001
	PGPR*AMF	1	24.47	<0.001	PGPR*AMF	1	3.07	0.082
Non-fluorescent colonies	AMF	1	32.48	<0.001	AMF	1	60.25	<0.001
	PGPR	1	134.01	<0.001	PGPR	1	209.04	<0.001
	PGPR*AMF	1	32.48	<0.001	PGPR*AMF	1	125.53	<0.001
**AVALON**								
	**14 days post inoculation**				**42 days post inoculation**			
	**Factor**	**d.f**.	**F**	**P**	**Factor**	**d.f**.	**F**	**P**
GFP-fluorescent colonies	AMF	1	0.06	0.807	AMF	1	15.09	<0.001
	PGPR	1	248.82	<0.001	PGPR	1	106.14	<0.001
	PGPR*AMF	1	0.06	0.807	PGPR*AMF	1	15.09	<0.001
Non-fluorescent colonies	AMF	1	1.72	0.192	AMF	1	2.87	0.095
	PGPR	1	26.08	<0.001	PGPR	1	32.81	<0.001
	PGPR*AMF	1	1.50	0.223	PGPR*AMF	1	2.87	0.095

Factor, independent variables (AMF, PGPR) and their interaction (PGPR*AMF); d.f., degrees of freedom; F, value for comparison with the critical value for significance; P, level of significance (P-value).

In comparison to ‘Mercato’, the rhizoplane of the cultivar ‘Avalon’ supported substantially lower colonization by GFP-expressing *P. putida* KT2440, but higher colonization by other (non-GFP expressing) culturable bacteria at 14 days (Fig. 2B, Table 1). Furthermore, bacterial colonization of the ‘Avalon’ rhizoplane was un-affected by co-colonization of *R. irregularis*. By 42 days, no substantial numbers of bacterial colonies (both GFP-expressing *P. putida* KT2440 and other culturable bacteria) could be isolated from the rhizoplane of ‘Avalon’.

### Plant development

**Figure 3:**
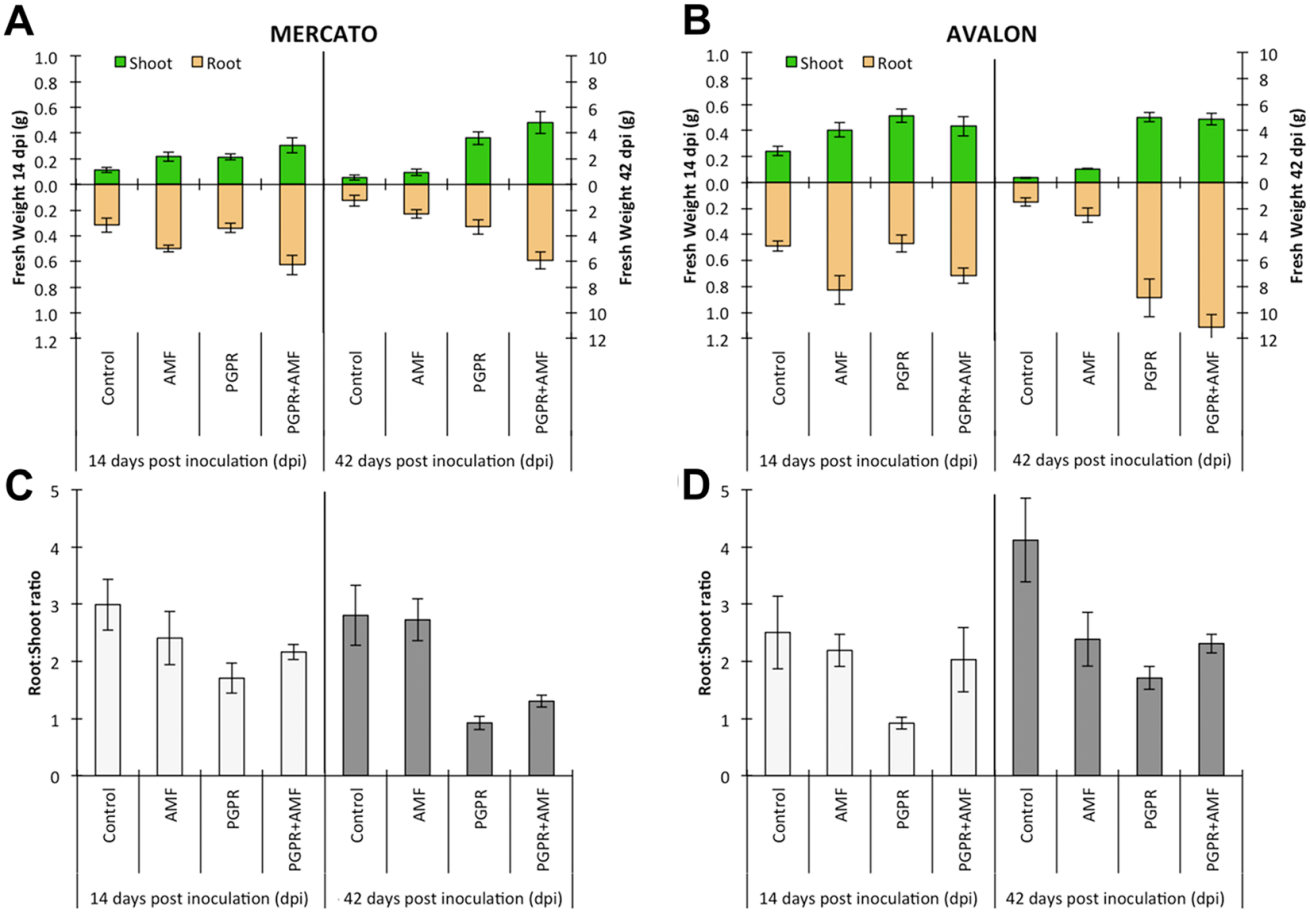
Fresh weight and root:shoot ratio of wheat with different inoculations. (**A**) Fresh weight of shoot (green) and root (orange) of ‘Mercato’ cultivar at 14 and 42 days post inoculation. Data at 14 days post inoculation are referred to left axis and data at 42 days post inoculation are referred to the right axis. (**B**) Idem as (**A**) for ‘Avalon’ cultivar. (**C**) Root:shoot ratio of ‘Mercato’ cultivar at 14 (white) and 42 (grey) days post inoculation. (**D**) Idem as (**C**) for ‘Avalon’ cultivar. Shown are average values (n = 10; ±standard error).

Inoculation with *R. irregularis* and/or *P. putida* KT2440 had a significant effect on the fresh weight (FW) of the shoots and roots and the root:shoot ratio of wheat plants, which varied both with time and wheat cultivar (Fig. 3, Table 2). Both *R. irregularis* and *P. putida* KT2440 increased shoot and root biomass at 14 and 42 days in ‘Mercato’ (Fig. 3A, Table 2). In ‘Avalon’ however, no significant effects of *R. irregularis* were seen at 14 days for shoots, but *P. putida* KT2440 caused a significant increase in shoot biomass (Fig. 3B, Table 2). For roots of ‘Avalon’, only *R. irregularis* caused a significant increase in biomass at 14 days. The combination of *R. irregularis* and *P. putida* KT2440 for both roots and shoots was additive at 14 and 42 days for ‘Mercato’. By contrast, ‘Avalon’ shoot biomass was supressed by the combination of *R. irregularis* and *P. putida* KT2440 relative to inoculation with *P. putida* KT2440 alone.

**Table 2:** Two-way ANOVA of fresh weight.

MERCATO								
	14 days post inoculation				42 days post inoculation			
	Factor	d.f.	F	P	Factor	d.f.	F	P
Leaves	AMF	1	9.43	0.006	AMF	1	4.17	0.056
	PGPR	1	9.42	0.006	PGPR	1	53.22	<0.001
	PGPR*AMF	1	1.22	0.283	PGPR*AMF	1	1.21	0.286
Roots	AMF	1	18.50	<0.001	AMF	1	9.04	0.008
	PGPR	1	1.50	0.236	PGPR	1	19.51	<0.001
	PGPR*AMF	1	0.09	0.767	PGPR*AMF	1	0.26	0.616
Roots:Shoot	AMF	1	0.11	0.744	AMF	1	1.04	0.321
	PGPR	1	5.40	0.031	PGPR	1	37.94	<0.001
	PGPR*AMF	1	1.84	0.191	PGPR*AMF	1	0.99	0.333
**AVALON**								
	**14 days post inoculation**				**42 days post inoculation**			
	**Factor**	**d.f**.	**F**	**P**	**Factor**	**d.f**.	**F**	**P**
Leaves	AMF	1	0.79	0.385	AMF	1	15.79	<0.001
	PGPR	1	6.18	0.022	PGPR	1	330.27	<0.001
	PGPR*AMF	1	4.78	0.041	PGPR*AMF	1	18.70	<0.001
Roots	AMF	1	17.98	<0.001	AMF	1	5.53	0.030
	PGPR	1	0.79	0.385	PGPR	1	87.08	<0.001
	PGPR*AMF	1	0.04	0.8452	PGPR*AMF	1	0.63	0.437
Roots:Shoot	AMF	1	2.10	0.163	AMF	1	0.21	0.650
	PGPR	1	6.92	0.016	PGPR	1	9.71	0.006
	PGPR*AMF	1	3.32	0.083	PGPR*AMF	1	5.44	<0.001

Factor, independent variables (AMF, PGPR) and their interaction (PGPR*AMF); d.f., degrees of freedom; F, value for comparison with the critical value for significance; P, level of significance (P-value).

The root:shoot ratio of ‘Mercato’, was significantly affected by the presence of *P. putida* KT2440 (Fig. 3C, Table 2). This was observed at both 14 and 42 days (Fig. 3C, Table 2). The same trend was observed in ‘Avalon’, at 14 days (Fig. 3D, Table 2). However, by 42 days, root:shoot ratio was significantly higher in plants co-infected with *R. irregularis* and *P. putida* KT2440 than plants inoculated with *P. putida* KT2440 alone. All treatments resulted in a lower root:shoot ratio than the control ‘Avalon’ plants at 42 days.

### Induction of callose deposition

**Figure 4:**
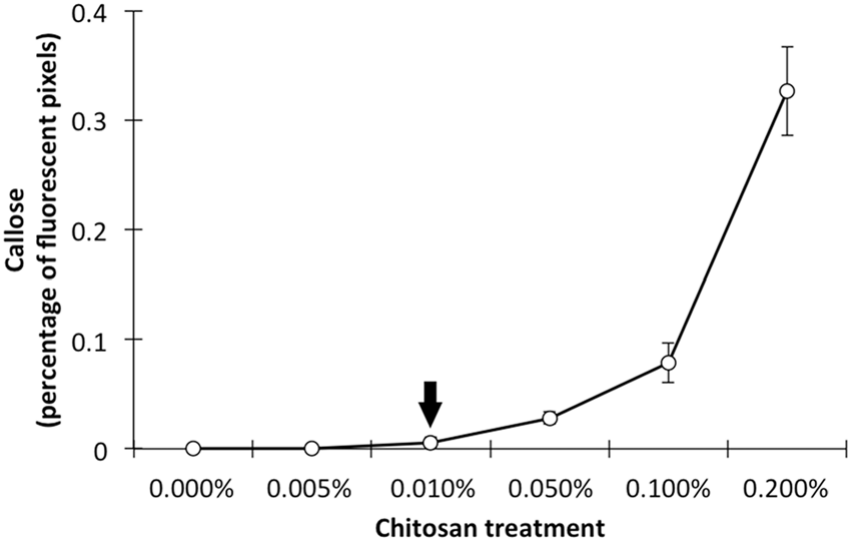
Calibration curve for identification of the optimal chitosan concentration (% w/v) not inducing callose deposition in un-primed wheat plants. Shown are average values (n = 10; ±standard error).

A calibration curve was developed prior to the experiment, in order to assess the optimal concentration of chitosan for measuring priming of callose deposition. Figure 4 shows that 0.01% (w/v) chitosan solution is the lowest concentration that does not induce callose deposition in un-primed plants, which was selected for further leaf infiltrations to test priming of callose deposition by microbial root inoculations.

**Figure 5:**
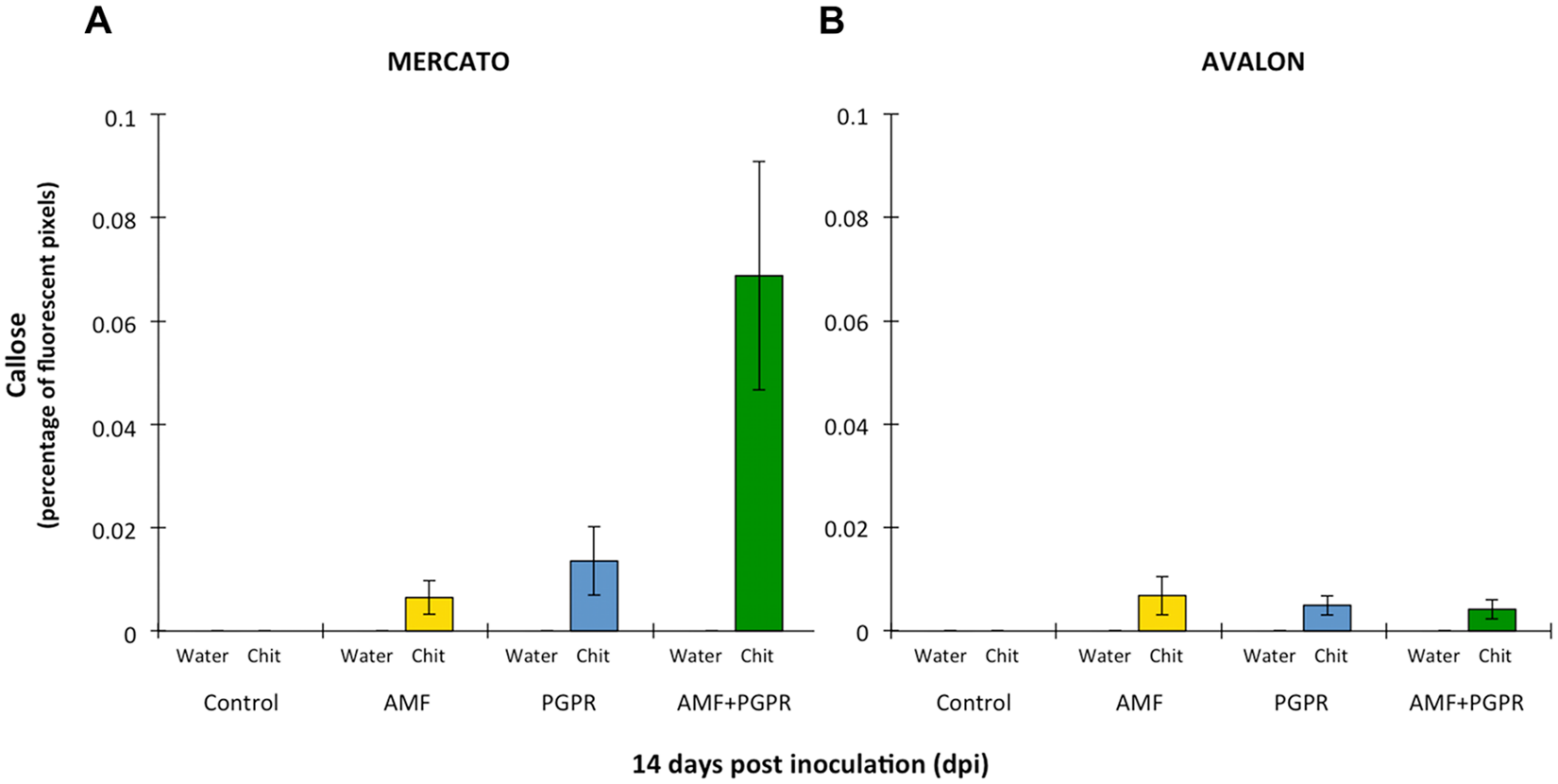
Induction of callose deposition in wheat leaves. (**A**) Percentage of fluorescent pixels of callose relative to the total number of plant material pixels in cultivar ‘Mercato’ at 14 days post inoculation. Callose deposition occurred only in leaves infiltrated with 0.01% chitosan, but not in buffer-infiltrated mock leaves. (**B**) Idem as (**A**) for cultivar ‘Avalon’. Shown are average values (n = 10; ±standard error).

Wheat cultivar and inoculation with *R. irregularis* and *P. putida* KT2440 influenced chitosan-induced callose deposition (Fig. 5, Table 3). At 14 days, infiltration of leaves with the buffer control solution did not induce callose deposition in any treatment of either ‘Mercato’ or ‘Avalon’. Similarly, infiltration with the relatively low concentration of 0.01% chitosan did not induce callose deposition in control-treated plants of either cultivar at 14 days. Chitosan treatment of leaves from *R. irregularis*-colonized and *P. putida* KT2440 colonised ‘Mercato’ showed relatively low levels of callose deposition, which was of comparable intensity. Interestingly, callose deposition in ‘Mercato’ that had been co-colonised by *R. irregularis* and *P. putida* KT2440 showed 10 and 5-fold higher levels of callose deposition compared to leaves from plants colonised by *R. irregularis* or *P. putida* KT2440 alone, respectively (Fig. 5A, Table 3). By contrast, co-colonised ‘Avalon’ plants showed similarly low levels of chitosan-induced callose as plants that had been colonised by *R. irregularis* or *P. putida* KT2440 alone (Fig. 5B, Table 3). At 42 days, infiltration with 0.01% chitosan failed to induce detectable levels of callose deposition for all cultivar-treatment combinations (data not shown).

**Table 3:** Two-way ANOVA of induction of callose deposition.

MERCATO				
	14 days post inoculation			
	Factor	d.f.	F	P
Callose (fluorescent pixels)	AMF	1	8.25	0.010
	PGPR	1	12.47	0.002
	PGPR*AMF	1	5.15	0.036
**AVALON**				
	**14 days post inoculation**			
	**Factor**	**d.f**.	**F**	**P**
Callose (fluorescent pixels)	AMF	1	1.78	0.197
	PGPR	1	0.26	0.615
	PGPR*AMF	1	2.79	0.111

Factor, independent variables (AMF, PGPR) and their interaction (PGPR*AMF); d.f., degrees of freedom; F, value for comparison with the critical value for significance; P, level of significance (P-value).

## Discussion

The rhizosphere and its associated microbiome are key drivers of the health and productivity of crop plants^8,29^. To understand the underlying mechanisms, we investigated the combined effects of a mycorrhizal fungus (*R. irregularis*) and plant growth-promoting rhizobacterium (*P. putida* KT2440) on immune responsiveness and above- and below-ground growth of two cultivars of wheat, ‘Mercato’ and ‘Avalon’ that differed in their ability to form mycorrhizal associations.

Both the AMF, *R. irregularis*, and the PGPR, *P. putida* KT2440, exerted a positive effect on wheat growth, however the two microorganisms differed in their relative effects on root and shoot biomass. In isolation, growth promotion by *R. irregularis* and *P. putida* is a well-characterised phenomenon, usually attributed to several factors, such as maximizing nutrient and water uptake in the case of AMF^7^, increasing availability of nutrients and/or production of phytohormones by PGPR^30^. In the absence of *P. putida*, and in line with previous reports^31^, *R. irregularis* did not change shoot growth relative to shoot root. In contrast to *R. irregularis*, but in agreement with previous studies of PGPR effects on root:shoot ratios^32^, root colonisation by *P. putida* in the absence *R. irregularis* led to preferential shoot growth, presumably as a reflection of a phytohormonal response related to increased root branching and more efficient nutrient uptake by plant roots. However, when plants were colonised by both AMF and PGPR, the root:shoot ratio was more similar to plants that had been inoculated with AMF only. This effect was similar for both cultivars at 14 and 42 days after inoculation. An intriguing result was the strong effect of PGPR on Avalon biomass despite the low bacterial abundance. A plausible explanation for this could again be related to the composition of root exudates, because such exudates are known to regulate bacterial gene expression^33^. For example, bacterial biosynthesis of the auxin indole-3-acetic acid (IAA) depends on the supply of exogenous tryptophan, and its presence in exudates from plant roots varies with genotype^34^. Consequently ‘Avalon’ and ‘Mercato’ may differ in the amount of compounds acting as precursors for the synthesis of plant hormones by the PGPR released in the rhizosphere, and thus affecting the behaviour of *P. putida* regarding plant growth promotion. This hypothesis warrants further investigation.

Based on our data, and in agreement with earlier studies on other plant species^8^, colonisation by both PGPR and AMF provides wheat with optimal growth conditions. It then follows that plants are capable of signalling to soil microbes, in order to facilitate a beneficial microbial community structure in the rhizosphere^10^. Until relatively recently, the chemical messengers deployed by plants into the rhizosphere remained poorly understood. A significant breakthrough came with the realisation that strigolactones induced branching in mycorrhizal hyphae and in so doing, enhancing root colonisation^14^. Strigolactones, coupled to reciprocal induction of MYC factors, lipochitooligosaccharides produced by the fungus that prime root morphology and chemistry for colonisation by AMF^35^, are now known to represent early regulators of AM symbiosis in nature.

Likewise, plants can produce chemical signals that elicit behavioural response of PGPRs^10,15^. For example, benzoxazinoid metabolites in root exudates of maize have been shown to induce positive chemotaxis in *P. putida* ^15^. Recent evidence suggests that benzoxazinoid production in the roots is enhanced by mycorrhizal infection^20,21^. Provided such changes in root benzoxazinoid composition result in enhanced exudation of these chemicals, they could promote a new equilibrium in microbiome composition^16,19^, commonly referred to as the “mycorrhizosphere effect”^10,18^. Based on this concept, we predicted that wheat plants colonised by AMF should attract greater numbers of resistance-inducing *P. putida* KT2440 than non-mycorrhizal plants.

Using GFP-tagged *P. putida* KT2440 as a marker for rhizoplane colonization by beneficial rhizobacteria, we recovered *circa* double the number of green-fluorescent CFUs from the rhizoplane of the cultivar ‘Mercato’ (which exhibited higher mycorrhizal colonisation) at 14 days, while this effect was not present in the cultivar ‘Avalon’ (which exhibited lower mycorrhizal colonisation). Other culturable rhizobacteria (e.g. non-GFP expressing) were also enhanced by the presence of mycorrhiza in ‘Mercato’ at 14 days. By 42 days, GFP-tagged *P. putida* numbers had declined in the rhizoplane of ‘Mercato’. This is unsurprising considering that benzoxazinoids are known to decline in root exudates of cereals with time post-germination^15,36^. Intriguingly, lower numbers of fluorescent CFUs were recovered from the rhizoplane of the less mycorrhizal cultivar ‘Avalon’ at 14 days. Furthermore, rhizoplane bacterial numbers from ‘Avalon’ dropped to almost undetectable levels by 42 days post-inoculation. Whether this is a function of a generic reduction in root exudates or, more specifically, a reduction in exudation of strigolactones and benzoxazinoids in ‘Avalon’ warrants further investigation. This question becomes especially relevant when considering that such specific changes in root exudation chemistry could account for the low mycorrhizal and low bacteria colonization phenotypes that we have observed in the ‘Avalon’ cultivar. It is also possible that ‘Avalon’ produces a water-soluble metabolite capable of inhibiting *P. putida* KT2440 growth. For example, catechin and compounds mimicking N-acyl homoserine lactone have been shown to interfere and inhibit bacterial quorum-sensing signals and hence, affecting biofilm formation and population density^37,38^.

Our rhizotron studies also revealed that *P. putida* significantly reduced arbuscule density in roots of ‘Mercato’. The mechanistic basis for this remains unclear. However, there are two distinct but mutually non-exclusive explanations: 1) *P. putida* is known to solubilise complex inorganic P (for example see Das *et al*.^39^), thereby increasing P supply to the host plant. It is also well established that high cellular P concentrations down-regulate strigolactone production^40^. This, in turn, would reduce AMF root colonisation, since hyphal-branching directly influences the degree of host colonisation by AMF^14,23^. 2) *P. putida* KT2440 has been shown to prime plant defences and induce systemic resistance^41–43^, which could dampen AMF infection. Successful establishment of the AM symbiosis is linked to down-regulation of local defences, most likely as a consequence of AMF effectors that are released during the early stages of the interaction. Since *P. putida* KT2440 colonization occurred before AMF colonization (Figs 1 and 2), it is plausible that the corresponding immune priming by *P. putida* antagonises the action of susceptibility-inducing effectors by AMF.

In addition to the effects of AMF and PGPR on rhizoplane colonization by other microbes and plant growth, AMF and PGPR can also systemically prime their host plants for augmented plant defence. This induced resistance cannot simply be attributed to improved plant nutrition as a result of AMF and PGPR colonisation of the roots^44^, but is rather a function of modulation of the host immune system by the symbionts^10,45^. This mycorrhiza-induced resistance (MIR) shares characteristics with pathogen-induced systemic-acquired resistance (SAR), such as priming of salicylic SA-dependent genes, and rhizobacterial induced systemic resistance (ISR), often characterized as priming of JA-dependent defences and cell wall defences^46^. This led Cameron *et al*.^10^ to propose that MIR might in fact be the additive product of AMF-induced and PGPR-induced priming mechanisms.

To test this hypothesis, we used callose deposition as a well-characterized marker for plant immune responsiveness to pathogen-associated molecular patterns, such as chitosan^47^. Callose, a β-glucan polysaccharide, facilitates reinforcement of the cell wall against attack and is thought to act as a matrix for the immobilisation of plant defence components, such as phytoalexins, reactive oxygen species, and cell-wall reinforcing enzymes (e.g. peroxidases)^47,48^. Leaf infiltration with 0.01% chitosan, which does not induce callose in un-primed plants (Fig. 4), resulted in detectable levels of callose deposition (quantified by epifluorescence) in both ‘Mercato’ and ‘Avalon’ at 14 days after inoculation with either *R. irregularis*, or *P. putida* KT2440. However, upon colonisation by both the AMF and PGPR, callose deposition in ‘Mercato’ was 10-fold greater than the AMF treatment alone, and 5-fold greater than the PGPR treatment alone. Hence, co-colonization by PGPR and AMF leads to synergistic levels of immune priming in AMF-responsive wheat. Conversely, callose deposition of co-inoculated ‘Avalon’ plants (which exhibited lower mycorrhizal colonisation) had no synergistic effect on callose deposition, suggesting fundamental differences in the signalling pathway leading to systemic immune priming. A threshold in PGPR population density has been shown as necessary for inducing effective resistance against phytopathogens^49^, so a minimum bacterial population could also be needed in order to synergistically prime the plant’s defences. Nevertheless, future work should be performed in order to completely validate this hypothesis using complementary techniques that allow identification of primed responses.

In conclusion, both AMF and PGPR can act additively on plant growth promotion, presumably due to complementary impacts on soil nutrient solubilisation and uptake. Moreover, co-colonization by AMF and PGPR appeared to have strongly synergistic effects on priming of host immunity, suggesting involvement of multiple defence pathways. This then supports a mechanistic explanation for the observations that MIR can be effective against both biotrophic and necrotrophic pathogens, which are resisted by different types of plant basal defences^50–52^.

## Materials and Methods

### Plant material and cultivation

We used two cultivars of wheat (*Triticum aestivum* L.) ‘Mercato’ and ‘Avalon’ that we had previously determined to differ in the extent of mycorrhizal colonisation seen in their root; Mercato exhibiting high colonisation levels and Avalon exhibiting low colonisation levels (and see results). Seeds were germinated in the dark at 20 °C and high humidity in between two rockwool blocks following the procedure described by Gurney *et al*.^53^. Seedlings were transferred after 7 days to rhizotrons built with 250 × 250 mm square petri dishes filled with sterile vermiculite, onto which was placed a 35 μm mesh (Plastok Associated, Birkenhead, UK)^53^. This kept the roots growing down between the mesh and the dish cover, allowing root observation during the experiment. Rhizotrons were covered with a black plastic sleeve for preventing light to reach the roots. Plants were grown in a controlled environment greenhouse room, with 12 h photoperiod and a day:night temperature of 20:18 °C. Rhizotrons were irrigated daily with 30 ml of 40% Long Ashton solution^54^ but lacking phosphorus. Fourteen days after sowing, plants were inoculated either with *in vitro* cultured spores of *Rhizophagus irregularis* isolate 09^55^, *Pseudomonas putida* KT2440 or both, leaving non-inoculated plants as control. Controls were treated with matching volumes of water and buffer. MgSO_4_ buffer does not contain P and MSR medium contains 0.41 g/l KH_2_PO_4_, so considering dilution with water and the rhizotrons volume, there were a final amount of 0.728 ppm of PO4 per rhizotron (available P in soil is usually around 20–50 ppm). At sampling dates (14 and 42 days post inoculation) fresh weight was determined for each plant. For further analysis and measurements, at least six plants per treatment and sampling date were used (totalling 96 plants).

### AMF species and cultivation

*Rhizophagus irregularis* isolate 09 was used as inoculum for AMF colonisation studies. The fungus was maintained and replicated using a monoxenic culture with transformed carrot (*Daucus carota* L.) roots according to the method established by Declerck *et al*.^56^. Infected roots were kept growing in 150 mm diameter petri dishes containing modified Strullu-Romand (MRS) agar medium. For inoculation of wheat plants in rhizotrons, the content of a petri dish was blended and mixed with 50 ml sterile distilled water. The concentration of spores was measured and adjusted to approximately 500–750 spores per ml, and 5 ml were added on the root of each plant in the rhizotrons.

### Bacterial strain and cultivation

A green fluorescent protein (GFP)-tagged *Pseudomonas putida* KT2440 strain previously described by Neal *et al*.^15^ was used for inoculation experiments. Stocks were stored at −80 °C and fresh cultures were used for inoculation. Bacteria were grown overnight in agitation (150 rpm) at 28 °C in M9 minimal salt medium supplemented with 0.1% glucose. Plants were inoculated with 3 ml of 10^8^–10^9^ bacterial suspensions in 10 mM MgSO_4_ buffer on the root surface.

### AMF and PGPR colonisation assays

Fourteen days after sowing, wheat plants were inoculated as described previously, giving four different treatments: non-inoculated control, AMF (*R. irregularis*) inoculated, PGPR (*P. putida* KT2440) inoculated, AMF and PGPR inoculated. Fourteen and forty-two days after inoculation (i.e. twenty-eight and fifty-six days after sowing), root samples were collected for assessing mycorrhizal and rhizobacterial colonisation.

For AMF quantification, root samples were fixed in 50% ethanol for at least 24 h at 4 °C. Then, roots were immersed in 10% KOH and subjected to an autoclave cycle (15 min, 121 °C, 15 psi), following stain with trypan blue during 20 min (0.4 g trypan blue + 50 g phenol + 50 ml lactic acid + 100 ml glycerol + 50 ml distilled water). After destaining 30 min in 50% glycerol, roots were washed, squashed and mounted on slides with 50% glycerol. At least ten fragments of 1 cm length were mounted on each slide for estimation of mycorrhizal colonisation according to Trouvelot *et al*.^28^ (see also Dodd *et al*.^57^). This method allows for the calculation of the frequency of colonisation of the root system (F) and arbuscule abundance in the root system (A).

Rhizobacteria colonisation was estimated by shaking root fragments during 20 min at 200 rpm in 10 mM MgSO_4_ buffer (10 ml per root g) and plating several dilutions onto petri dishes containing M9 minimal salt medium supplemented with 0.1% glucose. Plates were incubated in darkness at 28 °C during 48 h. Total number of GFP-expressing *P. putida* KT2440 colonies were determined using a transilluminator, whereas the other non-fluorescent bacteria colonies were counted under natural light. Data were expressed as number of colony forming units (CFU) per g of root fresh weight.

### Callose quantification

Fourteen and forty-two days after inoculation, samples from the youngest expanded leaf were collected from every plant for quantification of callose deposition. Leaf segments (2 cm) were infiltrated with 0.01% chitosan solution in 0.2% acetic acid (pH 5.7), and mock treatments with 0.2% acetic acid solution only. Vacuum infiltration was performed at −60 kPa for 5 min, and then the leaf segments were incubated on moistened filter paper in sealed petri dishes and collected for callose staining 24 h after infiltration. Leaf segments were fixed and destained in 100% ethanol at 4 °C for at least 48 h until tissue turned translucent. Then, they were washed and incubated in 0.07 M phosphate buffer (pH = 9) for 30 minutes, and finally stained overnight in darkness with 0.05% aniline blue in phosphate buffer. Samples were observed under epifluorescence (UV excitation: 330–385 nm; emission: 420 nm) using a BX51 Olympus microscope (Olympus Optical Ltd, London, UK) and pictures were taken with a connected DP71 digital camera. Callose was quantified by the number of deposition pixels relative to the total number of plant material pixels, using GIMP 2.8 software.

### Statistical analysis

Differences between treatments with microorganisms were analysed with two-ways ANOVA considering two factors (AMF inoculation and PGPR inoculation) using Statistix software (version 8.1). Variables were log-transformed when necessary to meet assumptions of normality and homogeneity. At least six replicates per treatment were used.

### Data availability

The datasets generated during and/or analysed during the current study are available from the corresponding author on reasonable request.
